# Serum Proteomic Profiling of Obsessive-Compulsive Disorder, Washing Subtype: A Preliminary Study

**DOI:** 10.18869/nirp.bcn.8.4.307

**Published:** 2017

**Authors:** Mona Zamanian-Azodi, Mostafa Rezaei-Tavirani, Naser Nejadi, Afsaneh Arefi Oskouie, Faird Zayeri, Mostafa Hamdieh, Akram Safaei, Majid Rezaei-Tavirani, Alireza Ahmadzadeh, Alireza Amouzandeh-Nobaveh, Farshad Okhovatian

**Affiliations:** 1. Student Research Committee, Shahid Beheshti University of Medical Sciences, Tehran, Iran.; 2. Proteomics Research Center, School of Allied Medical Sciences, Shahid Beheshti University of Medical Sciences, Tehran, Iran.; 3. Department of Biochemistry, School of Medicine, Tehran University of Medical Sciences, Tehran, Iran.; 4. Taleghani Hospital, School of Medicine, Shahid Beheshti University of Medical Sciences, Tehran, Iran.; 5. Department of Surgery, School of Medicine, Iran University of Medical Sciences, Tehran, Iran.; 6. Department of Microbiology, School of Medicine, Arak University of Medical Sciences, Arak, Iran.; 7. Physiotherapy Research Center, Shahid Beheshti University of Medical Sciences, Tehran, Iran.

**Keywords:** Obsessive-compulsive disorder, Washing subtype, Serum, Proteomics, Protein-Protein interaction network analysis

## Abstract

**Introduction:**

Obsessive-Compulsive Disorder (OCD) is a disabling mental condition that its proteomic profiling is not yet investigated. Proteomics is a valuable tool to discover biomarker approaches. It can be helpful to detect protein expression changes in complex disorders such as OCD.

**Methods:**

Here, by the application of 2D gel electrophoresis (2DE), a pilot study of serum proteome profile of females with washing subtype of OCD was performed. Serum samples were obtained from females with washing subtype of OCD. Following the protein extraction from the serum with acetone perception, the samples were subjected to 2DE for separation based on pI and molecular weight (MW) with triple replications. Finally, the protein spots were visualized using Coomassie blue staining method and analyzed by Progenesis SameSpots software. Furthermore, protein-protein interaction (PPI) network analysis was handled by the application of Cytoscape software.

**Results:**

The results suggested that 41 matched spots demonstrated significant expression alterations among which 5 proteins including immunoglobulin heavy constant alpha-1 (IGHA1), apolipoprotein A-4 (APOA4), haptoglobin (HP), protein α-1-antitrypsin (SERPINA1), and component 3 (C3) were identified by database query. Additionally, PPI network analysis indicated the central role of SERPINA1 and C3 in the network integrity. However, albumin (ALB), amyloid precursor protein (APP), and protein α-1-antitrypsin (APOA1) proteins were important in OCD PPI network as well. The identified proteins were related to 3 processes: acute-phase response, hydrogen peroxide catabolic process, and regulation of triglyceride metabolic process.

**Conclusion:**

It was concluded that these proteins may have a fundamental role in OCD pathogenesis. Moreover, the dysregulation of inflammatory and antioxidant systems in OCD risk was suggested by the current study. However, evaluation of bigger sample sizes and application of mass spectrometry are essential requirements to confirm this preliminary evaluation.

## 1. Introduction

Obsessive-Compulsive Disorder (OCD) is a complex neuropsychological condition with a lifetime prevalence of 1% to 2.5% in the general population (Campos, Yoshimi, Simão, Torresan, & Torres, 2015). The World Health Organization (WHO) introduced OCD as a leading cause of non-fatal illness-related disability that primarily affects people with the age range of 15 to 44 years (Zilhão et al., 2015). This anxiety disorder is characterized by intrusive thoughts and compulsive behaviors (Carmi, Dar, Zohar, & Zangen, 2015). Dysfunction in corticostriatal circuits is the main known implicated part of brain in pathology of OCD and OC-spectrum disorders (Burguiere, Monteiro, Mallet, Feng, & Graybiel, 2015).

The complex nature of this heterogeneous disorder is described by the influence of molecular and environmental interactions (Jaffe et al., 2014). That is, the combination of many factors is responsible for manifestation of this disorder with different subtypes. The contribution of many genes with their polymorphisms expresses different subtypes with overlapped symptom dimensions. This complex clinical feature of OCD is accompanied by other related disorders (comorbidity), including Tourette syndrome, chronic hair pulling, trichotillomania, and anxiety (Stewart, Jenike, & Keuthen, 2005). The complexity of OCD features made it challenging for treatment options. Most of the molecular studies in OCD are focused on genetic concept and genome-wide association studies of the disorder (Lin, Cao, & Gao, 2015; Zamanian-Azodi et al., 2015).

However, the pathophysiological origin of mental disorders, such as OCD, is still elusive despite these investigations. On the other hand, identification of the key relevant proteins in the disease condition is valuable for substantial insight of psychiatric disorders. By understanding etiological biomarkers of the disease, the underling mechanisms can be also explained (Filiou, Turck, & Martins-de-Souza, 2010). Body fluids are very useful sources to detect the biomarker; 1 of the appropriate ones is serum. Serum contains the vast range of proteins originated from normal or abnormal function of cells and tissue processes (Samavat et al., 2015; Tang, Beer, & Speicher, 2011). Moreover, serum is a suitable source (easily sampling and preparation) for different kinds of investigations as well as proteomics. Proteomic studies are important to determine expression levels of the proteins (Zali, Zamanian-Azodi, Tavirani, & Baghban, 2015). It is a powerful tool that provides useful information besides the other molecular studies (Rezaei-Tavirani et al., 2016). As mentioned earlier, OCD has various subtype models. One of the common models for the females is the washing compulsion (Noshirvani, Kasvikis, Marks, Tsakiris, & Monteiro, 1991). Proteins and mechanisms that correspond to the risk of this subtype are yet to be studied. To the authors‘ best knowledge, the current study was the 1^st^ proteome analysis of obsessive-compulsive disorder. In the current pilot study, the proteome profile of patients with OCD washing phenotype was studied by 2D electrophoresis and bioinformatics.

## 2. Methods

### 2.1. Sample collection

#### 2.1.1. Human subjects

The washing model cases (5 female patients) with moderate severity and control group (5 healthy volunteers) from Taleghani Hospital, Tehran, Iran, were diagnosed based on the diagnostic and statistical manual of mental disorders, 5^th^ edition (DSM-5). The cases signed written informed consents, and the control and case groups were demographically matched. The proteome of these pooled medication-free patients was compared with that of the pooled controls.

#### 2.1.2. Sample preparation

Blood collection was handled by venipuncture rout and 20-gauge needle. The samples were kept at room temperature for 30 minutes. After clotting, serum samples were separated by 2 times centrifugation at 2000g for 10 minutes at 4°C. Finally, the samples were maintained at −80°C for future processes (Nejadi et al., 2015). The protein extraction was conducted by the acetone precipitation method, according to introduction of Sigma ProteoPrep Protein Precipitation Kit.

#### 2.1.3. Proteomic experiment

All 2DE chemicals and Ready Strip™ IPG strips were provided by GE Health Life Science. Prior to 2DE, the total protein concentration of the samples was determined by Bradford assay. The 2DE procedure was handled with 3 replications of normal and OCD samples. The 1^st^ dimension, isoelectric focusing (IEF) was carried out by the application of Bio-Rad Protein IEF Cell, 7cm nonlinear IPG with the pH range of 3 to 11. In this step, about 500μg protein was loaded for each gel. The IEF separates proteins based on their pI. In the 2^nd^ dimension, proteins were separated based on Molecular Weight (MW) by 12% sodium dodecyl sulfate polyacrylamide gel electrophoresis (SDS-PAGE) gel and buffering systems in electrophoresis tank (Bio-Rad). After electrophoresis, the gels were dyed by Coomassie blue stating method and, then, scanned using Bio-Rad scanner (Hasanzadeh, Rezaie-Tavirani, Seyyedi, & Emadi, 2015). Finally, Progenesis SameSpots software was employed to analyze protein expression changes. A value of 1.5-fold increase or decrease was used as a cut off. Statistically significant differences (P≤0.05) in spot intensities were identified using 1-way ANOVA analysis. The significantly altered proteins in the expression were identified by *http://world-2dpage.expasy.org/swiss-2dpage/*. The amounts of MW and pI were the indices to identify proteins from this database.

### 2.2. Network analysis

Further investigation, based on interaction analysis, was carried out by Cytoscape 3.4.0-Milestone 2 software (Bader & Hogue, 2003). STRING database (DB) was the interaction source for PPI analysis by Cytoscape. STRING has the feature of providing comprehensive information from both experimental and predicted interactions of different databases with a probabilistic confidence score (Szklarczyk et al., 2010). Proteins identified by 2DE experiment were searched through STRING DB integrated in Cytoscape. The protein names were the query inputs and the selected species for this query was Homo sapiens as available in the query box.

The confidence (score) cut off for interactions was set to 0.5. Furthermore, about 100 additional nodes were added to the current study investigated proteins to expand the network. Network Analyzer plug-in analyzed functional topological parameters (centrality) including node degree and betweenness centrality (BC). This application is a powerful software to examine biological networks for the centrality parameters calculation by resourceful graph algorithms (Assenov, Ramírez, Schel-horn, Lengauer, & Albrecht, 2007).

Nodes that have high degree and betweenness values are the hub-bottleneck elements (Rezaei-Tavirani et al., 2016). MCODE, a Cytoscape Plug-in, examines protein complexes (regions with high interconnections) in PPI network. This Cytoscape plug-in is a clustering algorithm that examines the modules in a PPI network. Highly connected parts are detected by identifying nodes (seed) that are locally dense, travel outward from it, find the local neighbors, and isolate them as a cluster. The clusters are ranked based on the interconnection scores. Another level of functional annotations can be introduced by identifying the protein clusters. That is, proteins in a specific cluster usually indicate similar annotations (Bader & Hogue, 2003).

Finally, functional annotations of the current study investigated proteins and the detected modules were analyzed by the application of ClueGO plug-in (Bindea et al., 2009). GlueGO2.1.7 and its extended tool CluePedia annoted genes for biological process (BP). ClueGO presents gene ontology ranging from general to very specific ones as groups of related terms with similar shared proteins. The linkage strength between the terms, calculated by kappa score, was 0 to 1 (Bindea, Galon, & Mlecnik, 2013; Bindea et al., 2009). The kappa score was set to 0.5 for BP analysis. Minimum and maximum levels of ontology were set to 3 and 8 as the default option, respectively. The P was also set to ≤0.05. The correction method for P≤0.05 was Bonferroni step down method. The enrichment/depletion test for the terms was set to 2-sided enrichment/depletion, based on hypergeometric.

## 3. Results

**Figure 1:**
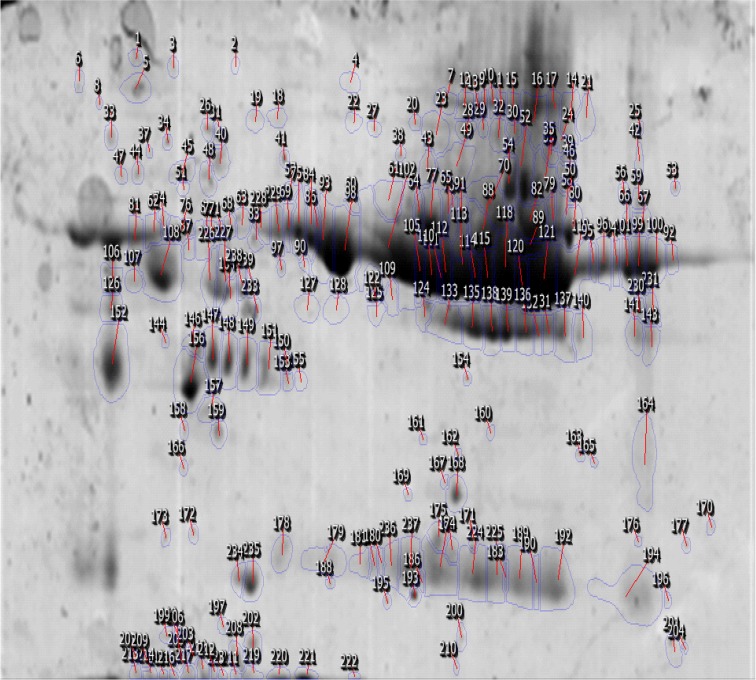
Position of matched spots on 2DE gel when serum sample with washing subtype and healthy controls were compared. The detected proteins are identified using SameSpots software.

**Figure 2:**
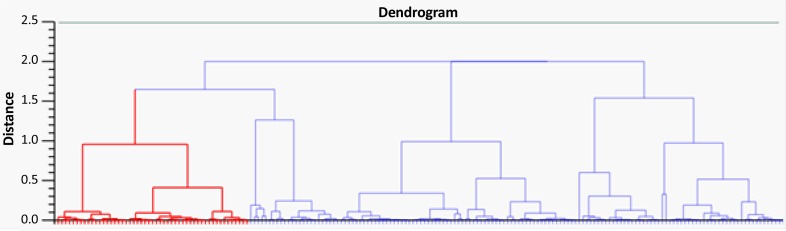
Three clusters with six sub-clusters of protein spots in OCD serum; the distance between the groups implied the expression difference levels. The dendrogram analysis was based on hierarchical clustering and automatic correlation analysis. The sub-clusters were defined by distance parameters less than 1.7. Sub-cluster1 in dendrogram was highlighted in red.

A total of 41 spots were differentially abundant in the OCD type that 18 showed upregulation and 23 downregulation. The abundant proteins, such as albumin and immunoglobulin, were not depleted in the current study. In Figure 1, positions of protein spots in this investigation are depicted. Progenesis SameSpots software revealed hierarchical clusters of proteins with similar expression correlations as dendrogram (Figure 2).

Five identified proteins with their properties are tabulated in Table 1. The codes and molecular functions of the elements were retrieved from Uniprot database. The position, MW and pI, and the condition of regulation of each specific protein in the samples obtained from patients with were found through the proteomic analysis.

**Table 1:** The significantly altered protein spots of serum samples obtained from patients with obsessive-compulsive disorder compared with the control group.

**Molecular Function**	**Condition**	**MW**	**pI**	**P**	**Fold Change**	**Protein ID**	**Protein Name**
Antigen binding	Downregulated	62433	5.61	0.05	−2	P01876	IGHA1
Antioxidant activity, cholesterol binding	Downregulated	10530	4.97	0.05	−3	P06727	APOA4
Protease binding	Downregulated	57706	4.91	0.01	−2.5	P01009	SERPINA1
Antioxidant activity	Downregulated	44344	4.88	0.02	−1.7	Q9UC67	HP
C5L2 anaphylatoxin chemotactic receptor binding, lipid binding	Upregulated	40915	4.84	0.05	1.5	P01024	Complement (C3)

The proteins were identified by *http://world-2dpage.expasy.org/swiss-2dpage/*. The amounts of MW and pI were the indices to identify proteins from this database. SameSpots software analyzed the position, MW and pI, and condition of regulation (up- or downregulation) of each specific protein in the samples of patients with OCD.

**Figure 3:**
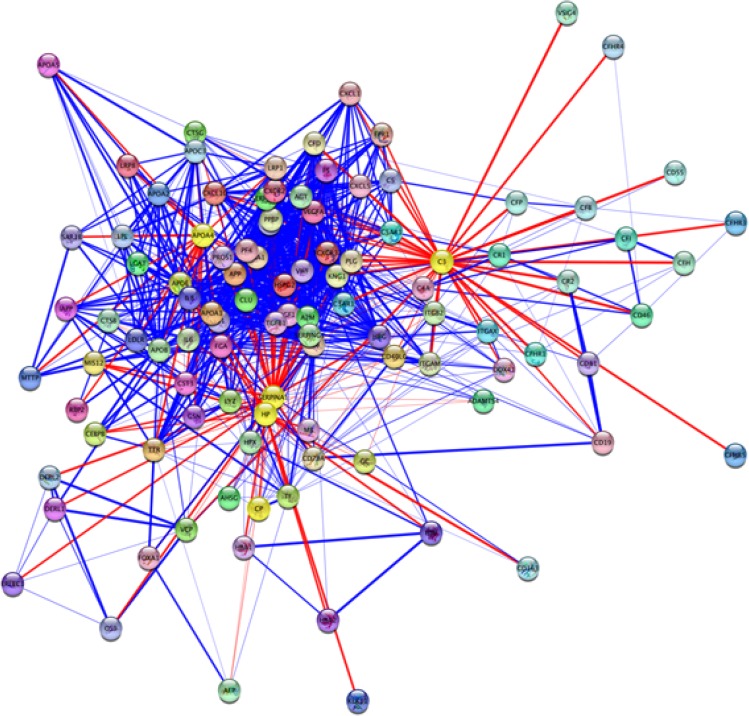
Protein-protein interaction network of 5 OCD related proteins from STRING-db source. The PPI network consists of 104 nodes and 885 edges. The cut off score for the network is 0.5. IGA1 is not retrieved in this network. The nodes highlighted in yellow are the query proteins. APOA4, HP, and SERPINA1 are connected directly (data are not shown).

PPI networks provide noteworthy information related to mechanisms of a specific disease (Safaei et al., 2016). Only 4 out of 5 proteins were involved in the current study PPI network. The interaction network was visualized by Cytoscape software version 3.4.0-Milestone 2 (Figure 3). Central properties of PPI network were analyzed using Network Analyzer application. Proteins with high degree and betweenness centrality scores were selected and presented (Table 2).

**Table 2:** List of hubs and bottlenecks.

**Central Proteins**	**Degree Score**	**BC Scores**
ALB	57	0.11
APP	47	0.048
C3	46	0.18
SERPINA1	46	0.09
APOA1	36	0.05

ALB: Albumin; APP: Amyloid Precursor Protein; C3: Complement 3; SERPINA1: protein α-1-antitrypsin; APOA1: Apolipo-protein A-1

**Figure 4:**
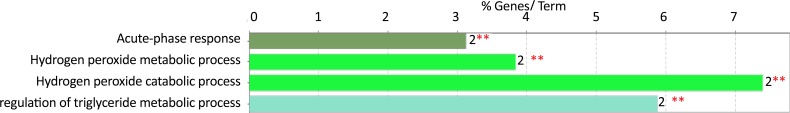
Four biological processes related to five identified proteins are presented. The number on the right side of each bar indicates the number of similar proteins in each term and the bars indicate the percentage (%Genes/Term). The (%Genes/Term) is higher in hydrogen peroxide catabolic process. Two star sign indicate statistically significant terms.

The proteins were ranked based on degree scores. Five central nodes were tabulated, out of which albumin showed the highest value. The C3 and SERPINA1 were the identified proteins in OCD gel sample and the 3 other proteins were imported from the network nodes. To determine the BP annotations of the selected proteins and investigated modules, ClueGO application was used to provide a better understanding of OCD pathophysiological processes (Table 3 and Figure 4).

**Table 3:** Biological processes annotations charts of five identified proteins. Identified differential proteins are related to three different groups and four different terms.

**Biological Process**	**Groups**	**Group Genes**
Acute-phase response	Group 0	HP|SERPINA1
Hydrogen peroxide catabolic process	Group 1	APOA4|HP
Regulation of triglyceride metabolic process	Group 2	APOA4|C3

HP, haptoglobin; SERPINA1, alpha-1-antitrypsin; APOA4, apolipoprotein A-4; C3, complement 3

The identified proteins were related to 3 different groups and 4 different terms. The number on the right side of each bar indicates the number of similar proteins in each term and the bars indicate the percentage (%Genes/Term). The (%Genes/Term) was higher in hydrogen peroxide catabolic process. Two-star sign is expressed the statistically significant values. The PPI network contained complex protein structures of densely connected regions. Protein complexes of OCD PPI network were retrieved by MCODE (Table 4).

**Table 4:** Detected clusters in the network and top-scored modules.

**Cluster**	**Topology of Module**	**Number of Nodes**	**Score**	**Seed**	**Proteins Presented in the Modules**
1	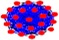	22	22	PF4	SERPINE1
2	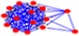	15	12.429	CXCL10	C3
3	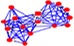	16	9.2	HSPG2	APOA4
4	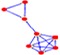	8	4	DERL1	-
5	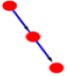	3	3	-	HP

HP: Haptoglobin; C3: Complement 3; APOA4: Apolipoprotein A-4

Eight clusters were detected in the network and 5 top-scored modules are presented. Cluster members are shown in red, and square nodes represent the seeds. Seed proteins are the important nodes with highest interaction score in the module.

**Figure 5:**
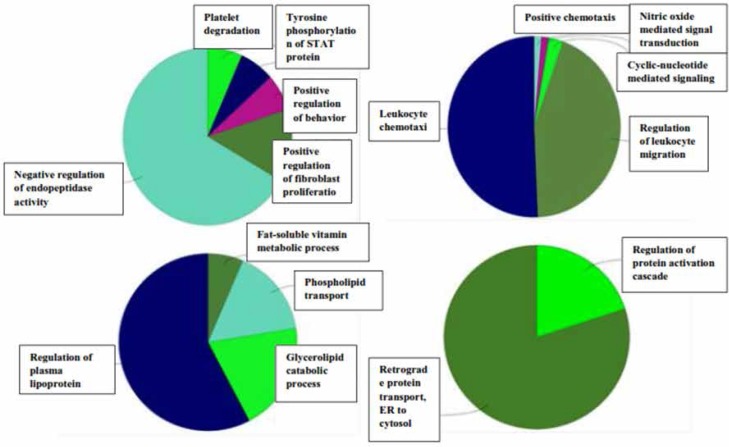
Biological process analysis of 4 top protein complexes; cluster 5 did not result in any BP groups with the defined parameters. The highlighted BPs in clusters 1 to 4 are the negative regulation of endopeptidase activity, leukocyte chemotaxis, regulation of plasma lipoprotein levels, and retrograde protein transport from ER to cytosol, respectively.

Seed proteins are PF4, CXCL10, HSPG2, and DERL1 for the clusters 1 to 4, respectively. SERPINE1, C3, APOA4, and HP are also present in the clusters 1 to 5, respectively. The statistical parameters of the analysis was based on the cutoff point of 2 and node score cutoff point of 0.2. Biological process analysis of four top clusters is presented in Figure 5.

## 4. Discussion

Molecular evaluation of obsessive-compulsive disorder is important due to the complex nature of the disorder. This feature resulted in an increased interest in the genetic studies. That is, most of the related molecular studies are primarily focused on genetic and genome concepts (Zamanian-Azodi et al., 2015). In this sense, researches show that many genes with related polymorphisms play key roles in the pathogenesis of OCD (Den Braber et al., 2016). On the other hand, proteomic study of OCD is not investigated yet. Proteomics can help to understand proteome changes of human during the disease state. In fact, many potential proteins may have expression alterations in this condition. The specific changed proteins are known as possible biomarkers that can facilitate diagnosis and treatment approaches in anxiety disorders. The current study analyzed proteins with significant expression changes in response to OCD pathogenesis. As shown in Figure 1, some protein spots in proteome profile of patient with OCD can have expression alterations. The presence of highly abundant proteins can have 2 opposite features. While it is possible to discover the feasible expression changes in these proteins, they can mask the detection of low abundance proteins (Nejadi et al., 2015).

In the current preliminary experiment, 1 of the main goals was to detect high abundant profile changes as well. Figure 2 indicates the clustering profile of all present proteins in OCD gel. Three clusters and 6 sub-clusters based on expression changes were obtained. The 1st sub-cluster is highlighted in red and the spots in each sub-cluster have close correlation values. The smaller the protein clusters, the bigger the correlation values. Proteins in each sub-cluster, especially those within smaller clusters, may show common ontology properties as their pattern of expression changes are similar. Detecting differentially expressed abundant serum proteins in OCD may provide information of other mechanisms of disorder development.

In most of the proteomic studies, these proteins are normally subjected to specific methods of depletion (Corbett et al., 2006). The possibility of contribution of these proteins in similar processes may be higher than that of the low-abundance proteins in serum. The reason for this claim is that low-abundance proteins are mostly secreted to the serum form different parts of the body and may be related to different pathways, while the main resident proteins of serum may be more correlated in functional properties. Observed proteins in the proteomic profile of patients with OCD, as indicated in Table 1, are IGHA1, APOA4, HP, SERPINA1, and C3.

There were significantly different expression patterns in the comparison of normal and patient samples. The first 4 proteins showed down-regulation, while C3 showed upregulation in the patient samples. Therefore, these proteins may have a central role in the disorder manifestation. Four of the proteins belonged to the 6 most abundant proteins in the serum including IgA, haptoglobin (HP), transferrin, and antitrypsin. Immunoglobulin heavy constant alpha (IGHA1) is a major immunoglobulin, here, the serum level of this protein changes significantly in the patients with OCD; however, it is not reported as a candidate protein in other mental disorders. APOA4, as a low abundant protein, belongs to the apolipoprotein superfamily. Apolipoproteins regulate the level of free fatty acids in plasma, high-density lipoprotein, and triglyceride-rich lipoproteins metabolisms, and have a role in the reverse cholesterol transport pathway (Levin et al., 2009).

Low expression of APOA4 may influence cholesterol level reduction in the serum of patients with OCD. This phenomenon may lead to dysregulation of neurons neurochemical (hyperactivity of serotonin reuptake receptor activity), as cholesterol is the main component of neurons (Madhura, 2015). Another function of APOA4 is as an antioxidant agent (Qin, Swertfeger, Zheng, Hui, & Tso, 1998); this role may justify the accumulation of oxidative product during OCD pathophysiology (Şimşek, Gençoğlan, & Yüksel, 2016). The downregulation of this protein is also reported in schizophrenia and the Alzheimer Disease (AD) (Levin et al., 2009; Lin, Cao, & Gao, 2015). HP is another serum abundant protein that binds to hemoglobin and has chaperone function. This protein shows downregulation in AD (Cocciolo et al., 2012).

Protein α-1-antitrypsin (SERPINA1) is the most abundant protease inhibitor in serum release from liver (Pastore, Ballabio, & Brunetti-Pierri, 2013). It shows down-regulation in OCD profile. This protein is also counted as a candidate biomarker in pathophysiology of schizophrenia (Davalieva, Kostovska, & Dwork, 2016). Complement C3 as a part of complement system is responsible for innate defense mechanism against pathogenic microorganisms (Ehrnthaller, Ignatius, Gebhard, & Huber-Lang, 2011). Increased level of C3 in the patients with OCD was observed in the current study. It may indicate that, immunity-related pathways are as part of the OCD underlying mechanism. Similar to neurodegenerative disorders, maintained activation of inflammation level in brain implies the protective effects to reduce toxic products of these brain disorders (Bonifati & Kishore, 2007).

In patients with schizophrenia, increased level of C3 level is also reported (Mayilyan, Weinberger, & Sim, 2008). Generation of oxidative products can be related to exaggerated or insufficient activation of the complement system (Bonifati & Kishore, 2007). Increment of oxidative products is reported in OCD (Şimşek et al., 2016). In fact, there are some overlaps between mental disorders and biomarkers of other neurological diseases. As mentioned above, expression alterations of APOA4, HP, and C3 proteins in OCD profile were also mentioned in other neurological diseases (Davalieva et al., 2016). Investigation of these proteins through PPI network can provide a better understanding of the prominent role of the detected proteins. The biological functions of identified proteins can be evaluated in this way (Levin et al., 2009). The expression changes of these proteins in serum may indicate integrative linkage of inflammatory response systems and the risk of OCD as it is also approved in other mental disorders such as schizophrenia (Davalieva et al., 2016).

In Figure 3, direct link among APOA4, HP, and SERPINA1 may specify an interaction among them. Network topology analysis showed central features of 2 proteins of C3 and SERPINA1 in the network as tabulated in Table 2. This analysis detected 3 proteins (ALB, APP, and APOA1) as candidates for central proteins that may be related to OCD risk. These proteins are also important in disorders such as schizophrenia (Huang, 2002; Levin et al., 2009; Yang et al., 2006). Biological processes related to differentially changed proteins may be remarkably influenced. As mentioned earlier, 1 of the important changes in brain diseases is the accumulation of oxidative products. This fact is also confirmed by gene ontology analysis of proteins identified in OCD shown in Table 3.

Hydrogen peroxide catabolic process is the primary-linked annotation of the identified proteins. The dysregulation of it may be one of the reasons of presentation of oxidative products. Further investigation of network topology identified 8 complexes of proteins, that the identified proteins in the current study are present in 4 of the top ranked ones. As shown in Table 4, central proteins in the network, SERPINA1 and C3, also belong to the 1st top clusters. This fact may imply the additionally important role of these proteins in the network integrity. The 5 top ranked protein complexes were further analyzed for biological process enrichment as depicted in Figure 5. The significant processes for the cluster 1 to 4 were the negative regulation of endopeptidase activity, leukocyte chemotaxis, regulation of plasma lipoprotein levels, and retrograde protein transport, ER to cytosol, respectively. These annotations may have important roles in OCD related mechanisms.

Finally, proteomic analysis and proteins identified in PPI network construction and their related processes were previously reported in other brain diseases as well. Consequently, literature review can be counted as an approach to validate the current preliminary study. Overall, the findings may be useful to understand molecular behavior of OCD and provide a starting point for further investigations of OCD proteome profile changes.

In conclusion, proteins involved in the OCD risk, provide new insight on the complexity of the disorder. IGHA1, APOA4, HP, SERPINA1, and C3 with significant expression changes, in particular the last 2 mentioned proteins with high centrality properties, may serve as potential treatment targets; however, furthered research is required to validate the preliminary information. It is also suggested to improve OCD diagnosis and treatment approaches by analyzing serum proteome with the focus on low-abundance proteins.
